# Editorial: Epigenetic and metabolic regulation of primary and metastatic brain cancers

**DOI:** 10.3389/fonc.2023.1271851

**Published:** 2023-09-07

**Authors:** Ryan C. Gimple, Briana C. Prager, Qi Xie

**Affiliations:** ^1^ Department of Medicine, Washington University School of Medicine, Washington University in St Louis, St. Louis, MO, United States; ^2^ Department of Neurosurgery, Massachusetts General Hospital, Boston, MA, United States; ^3^ Key Laboratory of Growth Regulation and Translational Research of Zhejiang Province, School of Life Sciences, Westlake University, Hangzhou, Zhejiang, China; ^4^ Westlake Laboratory of Life Sciences and Biomedicine, School of Life Sciences, Westlake University, Hangzhou, Zhejiang, China; ^5^ Institute of Basic Medical Sciences, Westlake Institute for Advanced Study, Hangzhou, Zhejiang, China

**Keywords:** brain cancer, cancer, epigenetics, metabolism, metastasis, tumor microenvironment, primary brain cancer, brain cancer stem cells

Primary intrinsic brain tumors and brain metastases are among the most lethal of all human malignancies despite decades of scientific advances. The intersecting fields of cancer epigenetics and metabolism lie at the core of our current conceptual framework for the pathophysiology of these diseases (1, 2). While alterations in epigenetic pathways contribute to neoplastic metabolic dysregulation through transcriptional control mechanisms, metabolite abundance and availability reciprocally impinge upon epigenetic pathways. In this Research Topic, we highlight important advances focused on epigenetic and metabolic processes that enable brain tumors to thrive within the intracranial setting, along with key insights into the tumor microenvironment.

Epigenetic pathways integrate multiple sources of information regarding cell state and microenvironmental features to orchestrate an organized cellular response, mediating cellular adaptability, plasticity, and resilience. In this Research Topic, McCornack et al. discuss mechanisms by which histone methyltransferases and acetyltransferases mediate these diverse processes including reviewing downstream signaling elements and therapeutic targeting opportunities. Valproic acid is a commonly used anti-epileptic with anti-tumor effects through its histone deacetylase (HDAC) activity; however, in this Research Topic, Barciszewska et al. define a new epigenetic role for valproic acid in the induction of global hypermethylation. This study provides a rationale for the combination of valproic acid with temozolomide for enhancing DNA damage and oxidative stress in glioblastoma tissues. Although extrachromosomal circular DNA (eccDNA) had been originally observed nearly six decades ago (3), the relevance of this alternative mechanism of dynamic gene regulation to cancer survival and therapeutic resilience has only recently come into sharper focus (4, 5), particularly in brain tumors (6–9). Zhu et al. interrogate the landscape of eccDNAs in medulloblastoma, showing preferential inclusion of genes involved in neuronal development and differentiation, RAS GTPase binding, and RAP1 signaling. They further identify the upregulation of genes via eccDNAs associated with poor prognosis in clinical datasets, highlighting eccDNAs as important mediators of medulloblastoma pathophysiology and implicating future therapeutic targets.

Tumor metabolism impinges on multiple key oncogenic processes through the coordination of signaling cascades, bioenergetics, and structural components both in a cell-intrinsic manner and in the coordination of cellular interactions within the brain tumor microenvironment. Bezawork-Geleta et al. review the importance of lipid metabolism in glioblastoma biology, focusing on lipid droplets as key substrates for cellular energy production and as intermediates that affect cellular signaling through affecting ferroptosis (a lipid peroxidation mediated and iron-dependent form of cell death), and lipophagy (a lipid specific form of autophagy). Autophagy is a finely tuned cellular process that enables cancer cell survival in nutrient- and energy-poor settings that define the tumor microenvironment, but can also be exploited for anti-cancer therapies (10). Induction of cytotoxic autophagy using the small molecule inhibitor ABTL0812 impairs glioblastoma stem cell proliferation and stem features *in vitro* and displays combinatorial efficacy in orthotopic xenograft models when combined with standard-of-care radiotherapy and temozolomide, primarily through inhibition of AKT/mTORC1 signaling and activation of ER stress responses (Mancini et al). These studies highlight the important metabolic adaptations utilized by primary brain tumors to survive in the intracranial setting and suggest a new approach to undermining these key dependencies.

Epigenetic and metabolic pathways further support tumor cell invasion. The blood-brain barrier preserves the integrity of the central nervous system and shields against toxins from the central circulation and from invading cancer cells. Breaching the blood-brain barrier is a critical step in the metastatic cascade responsible for the generation of brain metastases (11). Zhang et al. identify that the blood-brain barriers of patients with advanced lung cancers are more permeable than those of patients with early-stage lung cancers or healthy controls, suggesting that primary lung cancers may act at a distance to disrupt the blood-brain barrier and set the stage for future metastases. Further understanding of the processes underlying blood-brain barrier disruption may yield strategies to protect the brain from metastatic colonization. Meningiomas are among the most common primary brain tumors with widely variable prognoses based on molecular classification and morphologic features. Jiang et al. utilize imaging characteristics from MRI studies to improve the identification of the extent of tumor invasion to facilitate improved extent of resection. This study further identifies imaging features that serve as independent risk factors for predicting WHO tumor grades through deeper scrutiny of interactions within the brain-tumor interface.

This Research Topic further touches on therapeutics in the intracranial setting. Vasogenic edema is a major source of morbidity and mortality in intracranial malignancies with limited treatment options (12). While studies have shown the efficacy of bevacizumab in reducing edema and progression-free survival in primary brain tumor patients (13, 14), Bai and Zhou investigate the role of bevacizumab in patients with lung or colon brain metastases, showing differential effectiveness based on the tumor of origin. The prognosis for patients with brain metastases from non-small cell lung cancer remains dismal, particularly when patients are not candidates for surgical resection due to a variety of patient or tumor characteristics. Yang et al. study the use of an alternative radiation therapy delivery modality, 125-Iodide brachytherapy, compared to external beam radiotherapy (EBRT) and find improved 6-month survival and similar 12-month survival for the 125-iodide group compared to the EBRT group. This study provides further evidence for an additional modality for the treatment of brain metastases.

In a prescient and well-timed review, Johanssen et al. describe the current state of basic and translational research as it applies to glioblastoma with a discussion of the importance of understanding brain tumors through the lens of intratumoral heterogeneity and within the context of their tumor microenvironments. They suggest the use of both rationally designed and hypothesis-generating unbiased combinatorial screening approaches that incorporate heterogeneous tumor models as well as important microenvironmental features to identify compounds and combination therapies with the greatest clinical utility. Taken together, this Research Topic highlights some of the latest advances in the fields of brain tumor biology, specifically focused on the intersection between cancer epigenetics and metabolism and touching on key elements of the tumor microenvironment, with an eye towards improving outcomes for patients with primary and metastatic brain tumors.

## Author contributions

RG: Writing – original draft, Writing – review & editing. BP: Writing – original draft, Writing – review & editing. QX: Writing – original draft, Writing – review & editing.
